# Gray and white matter integrity influence TMS signal propagation: a multimodal evaluation in cocaine-dependent individuals

**DOI:** 10.1038/s41598-018-21634-0

**Published:** 2018-02-19

**Authors:** Tonisha E. Kearney-Ramos, Daniel H. Lench, Michaela Hoffman, Brittany Correia, Logan T. Dowdle, Colleen A. Hanlon

**Affiliations:** 1 Department of Psychiatry, Medical University of South Carolina, Charleston, South Carolina USA; 2 Department of Neurosciences, Medical University of South Carolina, Charleston, South Carolina USA; 3 Center for Biomedical Imaging, Medical University of South Carolina, Charleston, South Carolina USA; 4 Ralph S. Johnson VA Medical Center, Charleston, South Carolina USA

## Abstract

Transcranial magnetic stimulation (TMS) can stimulate cortical and subcortical brain regions. However, in order to reach subcortical targets, intact monosynaptic connections are required. The goal of this investigation was to evaluate the contribution of white matter integrity and gray matter volume to frontal pole TMS-evoked striatal activity in a large cohort of chronic cocaine users. 49 cocaine users received single pulses of TMS to the frontal pole while BOLD data were acquired – a technique known as interleaved TMS/fMRI. Diffusion tensor imaging and voxel-based morphometry were used to quantify white matter integrity and gray matter volume (GMV), respectively. Stepwise regression was used to evaluate the contribution of clinical and demographic variables to TMS-evoked BOLD. Consistent with previous studies, frontal pole TMS evoked activity in striatum and salience circuitry. The size of the TMS-evoked response was related to fractional anisotropy between the frontal pole and putamen and GMV in the left frontal pole and left ACC. This is the first study to demonstrate that the effect of TMS on subcortical activity is dependent upon the structural integrity of the brain. These data suggest that these structural neuroimaging data types are biomarkers for TMS-induced mobilization of the striatum.

## Introduction

Transcranial magnetic stimulation (TMS) is an FDA-approved technique for treating depression and migraine, and is being used in clinical trials for a number of neuropsychiatric disorders including addiction, pain, and stroke rehabilitation. One of the powerful aspects of TMS is that it has the ability to change activity in the cortical site directly targeted by the coil as well as in subcortical afferent targets. This was demonstrated elegantly by Strafella and colleagues, who showed that dorsolateral prefrontal cortex TMS can cause an increase or decrease in caudate dopamine^1^. Numerous studies have now demonstrated that it is possible to induce causal changes in the striatum, the cingulate, and the insula by applying TMS with a figure-of-eight coil to the ventromedial medial prefrontal cortex (VMPFC, or frontal pole)^2–5^.

An often-overlooked element, however, is that subcortical modulation via TMS requires that the induced electric field at the cortex be able to reach the subcortical targets through intact structural connections. Given that numerous patient populations with neurologic and psychiatric disorders have low gray matter volume and disrupted white matter integrity, these structural features of the brain may be very important biomarkers of TMS efficacy. To date, however, very little is known about the contribution of gray matter volume and white matter tract integrity to subcortical modulation in patients.

The primary goal of this study was to evaluate the effect of gray matter volume and white matter integrity on striatal engagement by TMS. This was done in a cohort of 49 cocaine-dependent individuals. The choice of cocaine-dependent individuals was motivated by two factors (1) a growing momentum to develop TMS as a treatment tool for this population and (2) a growing knowledge that both gray and white matter deficits are present in cocaine users and are related to treatment outcome in typical behavioral treatment programs^6,7^. This experiment evaluated the impact of two domains of neural structure on TMS signal propagation: (1) the contribution of gray matter volume at the site of stimulation and in the primary afferent targets of the ventromedial prefrontal cortex and (2) the white matter integrity along the pathway between these regions (average fractional anisotropy). Demographic and drug use variables were also evaluated as factors associated with TMS signal propagation variability.

## Methods

### Participants

#### Recruitment and Selection Criteria

All experimental protocols were reviewed and approved by the Medical University of South Carolina Institutional Review Board (MUSC IRB). All study methods were carried out in accordance with relevant guidelines and regulations. Fifty-one (51) non-treatment-seeking cocaine-dependent individuals [26 males, 25 females; mean (SD) age = 38 (9) years] were recruited from the community to participate in one of three multimodal imaging studies. The participants were recruited through treatment programs and had to have used cocaine or crack-cocaine by any route within the last 5 years. Potential participants were excluded if they reported current use of prescription or illicit psychoactive drugs other than cocaine or marijuana, or if they reported more than 100 lifetime uses of any drug of abuse other than cocaine. Additional exclusion criteria include a score of more than 15 on the AUDIT^8^, smoking >1 pack of cigarettes per day, current breath alcohol concentration >0.002, a lifetime history of head injury with loss of consciousness, being pregnant or breast feeding, unstable medical illness, or a current DSM-IV Axis I psychiatric disorder.

#### Clinical Assessments

Informed consent procedures were approved by the MUSC IRB and obtained from each participant. Following informed consent procedures, participants completed screening assessments related to protocol safety, medical, and psychiatric history. Clinical and drug use history self-report assessments included the Structured Clinical Interview for DSM-IV (SCID^9^); Timeline Follow-back (TLFB; for cocaine, alcohol, marijuana, and nicotine^10^; Alcohol Use Disorders Identification Test (AUDIT^8^); Fagerström Smoking Inventory^11^; Beck’s Depression Inventory (BDI-II^12^); and Spielberger State-Trait Anxiety Inventory (STAI^13^).

### Stimulation Protocol and Data Acquisition

#### TMS Motor Threshold

Participants were brought into the imaging center to determine their resting motor threshold—rMT, the minimum stimulation intensity needed to apply over the hand area of the primary motor cortex to induce contraction of the contralateral abductor pollicis brevis muscle at least 50% of the time—using the standardized PEST procedure^14^. The resulting rMT parameters were used to set the stimulation dose for the TMS/fMRI session. During the motor threshold process, TMS was applied using a Magstim SuperRapid stimulator (Magstim Inc., Morrisville, NC) which generates biphasic electrical pulses (250 µs). The stimulator was located outside of the MR scanning room and the cable which is typically attached to the TMS coil was attached to an RF filter. The pulses were delivered through an 8-m cable which was attached to the bottom of an RF filter and passed through the waveguide into the MR scanning room where it was led through the bore of the MRI scanner and terminated in a custom non-ferromagnetic figure-of-eight TMS coil.

#### TMS Coil Positioning

**Figure 1:**
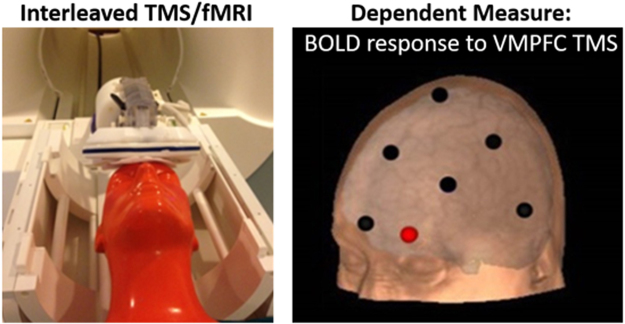
Experimental Design. TMS was delivered to the left frontal pole in 51 individuals with cocaine use disorder. The TMS pulses were interleaved with T2*-weighted functional imaging (left). The position of the coil was based on the EEG 10–20 system FP1 location (right).

Consistent with our previous TMS studies^3–7^, VMPFC coil position was determined using standardized coordinates from the EEG International 10–20 system for electrode placement (with FP1 corresponding to the left VMPFC stimulation target; Fig. 1). The EEG 10–20 system was used as the basis for TMS coil positioning as it accounts for variability in participant skull size and is consistently used in clinical TMS applications. The location and orientation of each participant’s coil placement was indicated on a nylon cap which participants wore throughout the study visit. Participants were positioned supine on the scanner bed and the TMS coil was mounted in the MR head coil with a custom TMS coil holder adjustable in six directions.

#### Interleaved TMS/fMRI Image Acquisition

All participants were scanned using a Siemens 3.0 T Tim Trio (Siemens Medical, Erlangen, Germany) MRI scanner with a 12-channel head coil. A series of single TMS pulses (100% rMT) were applied during a 100-ms gap between volumes (flip angle = 90°; TR/TE = 2500 ms/23 ms; FOV = 192 mm; 43 slices; final voxel resolution = 3mm^3^). Pulses were presented every 10–12 sec. For the three data sets included in this investigation, the total volumes collected were 35, 106, and 44, respectively. The number of pulses administered was included as a covariate in all analyses to account for differences in TMS/fMRI parameters across the three data sets.

#### Diffusion Tensor Imaging

Diffusion-weighted images were obtained using a twice-refocused echo-planar sequence with 2 diffusion weightings (b = 0, 1000 s/mm^2^) along 30 diffusion encoding directions (50 slices, 0% distance factor, 222 × 222 FOV, 74 × 74 matrix, TR = 6400 ms, TE = 96 ms, slice thickness = 2.7 mm, partial Fourier encoding: 6/8, no interpolation, 2 averages). Forty (40) subjects had diffusion images. Eighteen of the 40 subjects had a diffusion protocol identical to the first set with the exception of the following parameters: TR = 6700 ms, TE = 87 ms, slice thickness = 3 mm.

#### Anatomical MRI Acquisition for Voxel-based Morphometry

High-resolution T1-weighted images were obtained using an inversion recovery 3D spoiled gradient echo (3DSPGR) sequence (TR = 1900ms, TE = 2.26 ms, flip angle = 90°, 128 slices, matrix size = 256 × 256, FOV = 24 cm, slice thickness = 1.5 mm with no gap between slices, giving an in-plane resolution of 0.94 mm).

### Neuroimaging Data Analysis

#### Interleaved TMS/fMRI Image Analysis

Standard spatial preprocessing was performed on TMS/fMRI data via SPM12 (Welcome Department of Cognitive Neurology, London, UK) implemented in Matlab 7.14 (MathWorks, Inc., Natick, MA) using custom scripts. The data were corrected for acquisition time (slice timing), realigned to the first volume (motion correction), normalized into a standardized neuroanatomical space (Montreal Neurological Institute (MNI) ICBM152 brain template), and smoothed using a Gaussian kernel of 8 mm for the group analysis to reduce the variance due to anatomical variability. Of the 51 recruited participants, 2 participants were excluded for excessive head motion artifact (>2 mm in any plane; X, Y, Z, roll, pitch, yaw). Data analyses were conducted on the remaining 49 participants [26 males, 24 females; mean (SD) age = 39 (9) years; see Table 1 for detailed demographics]. General linear modeling (GLM) analysis of fMRI data was performed for each participant modeling the TMS pulse as an event convolved with the canonical hemodynamic response function. Statistical contrast maps were constructed for each participant by comparing brain response to the TMS pulses vs. brain activity during baseline (rest periods). A group-level contrast map was then computed to reveal significant areas of TMS-induced brain activation across participants. Significant clusters were identified as those with two-sided p < 0.001, uncorrected, k = 58 to achieve cluster-level p-values at a Family Wise Error (FWE)-correction equivalent to α < 0.05 (SPM12 default uses Gaussian Random Field Theory correction).

**Table 1:** Descriptive demographic, clinical, and drug use statistics.

	**n** = **49** Total sample
**Demographics**	
Sex	26 M, 23 F
Age	38.5 (±8.9) years
Ethnicity	35 AA, 13 C, 1 H
Education	12.8 (±2.0) years
**Cocaine use**	
Preferred drug administration	19 smoke, 19 snort, 3 both, 8 unreported
Age of first cocaine use	21.5 (±6.4) years
Total duration of cocaine use	17.1 (±9.4) years
Amount $ spent per week	$187.24 (±$172.36)
Days used in last 30 days	11.8 (±7.4) days
Time since last use (at visit)	6.1 (±16.3) days
**Other substance use**	
Nicotine smokers	43 (88%)
Nicotine severity (Fagerström)	2.9 (±2.5)
Marijuana smokers	35 (71%)
Days MJ used in last 30 days	7.4 (±10.5) days
Alcohol use severity (AUDIT)	9.3 (±7.0)
Age first alcohol use	16.4 (±3.4) years
**Mental status**	
Depressive symptoms (BDI)	8.7 (±9.1)
State Anxiety (STAI-S)	33.9 (±12.7)
Trait Anxiety (STAI-T)	38.1 (±12.8)
**TMS-related measures**	
Scalp-to-cortex distance (mm)^¥^	16.2 (±3.3) mm
TMS threshold^%^	72.8% (±12.8%)

Abbreviations: M = male; F = female; AA = African-American; C = Caucasian; H = Hispanic; MJ = marijuana; AUDIT = Alcohol Use Disorders Identification Test; BDI = Beck’s Depression Inventory; STAI = Spielberger State-Trait Anxiety Inventory. Values either indicate mean (±standard deviation) or count (percent^%^). ^¥^Scalp-to-cortex distance (mm) for VMPFC coil placement at EEG 10–20 FP1.

#### Diffusion Tensor Imaging Analysis

Raw diffusion-weighted images (DWIs) were first quality checked for artifacts and excessive head motion. FSL’s v5.0 (https://fsl.fmrib.ox.ac.uk/fsl/fslwiki/) BET was used to extract the brain from each subject’s B0 image. DWI’s (1 B0, 30 gradient directions) were then preprocessed using FSL’s eddy, and masked with their respective B0 extracted brain. Motion-corrected DWIs were then imported into MRtrix3 (http://www.mrtrix.org) to estimate tensors. Fractional anisotropy (FA) and mean diffusivity (MD) metrics were then calculated^15^. Using FSL’s Tract Based Spatial Statistics, FA maps were normalized into MNI space and skeletonized. Tract ROIs were then overlaid on all subjects’ skeletonized FA or MD maps and metric values were extracted.

Tract ROIs were created using the WU-Minn Human Connectome Project (HCP)−842 atlas which contains reconstructed diffusion data in MNI space (http://dsi-studio.labsolver.org/download-images/hcp-842-template)^16^. Fibers from the HCP-842 (2 mm) atlas were tracked (DSI Studio, angular threshold 60 degrees) by placing a seed in FP1 and end regions in 8 regions of interest from the standard AAL template that showed significant activation following VMPFC stimulation (right and left caudate, putamen, ACC, and insula) (see Supplemental Figure S1). Tracts identified using the HCP-842 atlas included FP1 to left caudate, left putamen, left ACC, and right ACC. Tracts were converted into ROIs for subsequent analysis. To determine the relationship between TMS-evoked BOLD response and white matter integrity, each participant’s FA and MD values were used as regressors to first-level contrast maps of interleaved TMS/fMRI. The analysis was performed as a GLM within SPM12. As previously described^5^, an implicit mask restricted analysis to regions shown to respond to TMS over the frontal pole including the frontal cortex, insula, amygdala, pallidum, thalamus, hippocampus, and striatum. Partial correlations of BOLD (beta weights extracted from significant clusters) and FA were performed using SPSS version 24. Covariates modeled in the GLM and partial correlations included age, number of interleaved TMS pulses administered, diffusion parameter group, and scalp-to-cortex distance.

#### Voxel-Based Morphometry

Voxel-based morphometry (VBM) was executed using SPM12. First, T1-weighted structural images were segmented into white matter (WM), gray matter (GM), and CSF images using SPM’s Segment tool. DARTEL was used to determine non-linear deformations needed to align subjects’ GM images^17^. DARTEL was utilized because it increases the accuracy of inter-subject registration, a feature critical to voxel based morphometry studies that allows for more precise comparisons of GMV across subjects^18^. Finally, images were spatially normalized, brought into MNI space and smoothed using a 6 mm FWHM kernel. Analyses were performed using “modulated” GM images. Local gray matter volume (GMV) estimates (measured as units of voxel intensity) were extracted from each subject’s left and right caudate, putamen, ACC, insula, and FP1 based on data from previous studies^5^). Regions-of-interest (ROIs) were selected from the standard Automated Anatomical Labeling (AAL) Atlas. (AAL: left caudate (AAL: Caudate_L), right caudate (AAL: Caudate_R), left putamen (AAL: Putamen_L), right putamen (AAL: Putamen_R), left insula (AAL: Insula_L), right insula (AAL: Insula_R), and anterior cingulate cortex (AAL: left and right Cingulum_Ant). Masks of each ROI were created in Matlab using the WFU PickAtlas toolbox (Wellcome Trust Centre for Neuroimaging, London, UK). Additionally a custom ROI was created for FP1 using a cortical location previously described^19^, wherein a spherical ROI was created with a radius of 20 mm and masked with an MNI brain mask. Global normalization was accounted for by dividing each ROI’s mean GMV value by the total intracranial volume (TIV) of that subject. To determine the relationship between TMS-evoked BOLD response and GMV, VBM GMV values from each subject were used as regressors to first-level contrast maps of interleaved TMS/fMRI. Partial correlations of BOLD (beta weights extracted from significant clusters) and GMV were performed using SPSS version 24.0. Control variables for the GLM and partial correlations included age, number of interleaved pulses administered, and scalp-to-cortex distance

#### Scalp-to-Cortex Distance Quantification

Given that effects of TMS on cortical depolarization are proportional to the distance between the scalp and cortex^20,21^, we calculated the distance from the scalp to cortex on the transverse plane of anatomical images for each participant using dedicated scalp-to-cortex distance measurement software^22^. The average distance from participant-specific placement of FP1 to the nearest cortex was 16 mm (±3 mm). These distances were incorporated into the analyses as covariates.

#### Stepwise multiple linear regression

To evaluate the contribution of clinical variables to variance in TMS-evoked BOLD signal, the *stepwiselm* function in MATLAB R2017b was used. This function uses both forward and backward steps to identify the best linear model for that data. The AIC was used as the criterion for inclusion. A separate model was built for each brain region, beginning with the same set of predictor variables. The initial set of variables included can be found in the first column of Supplemental Table S1, all were selected for practical potential and to limit multicollinearity. Of the full potential set of clinical variables, trait anxiety was omitted, due to its high relation to state anxiety, and participants age was not included because it was a strong predictor of years of use. The stepwise linear regression method is associated with some limitations, particularly when it comes to interpreting the usefulness of the final model. For this reason, we choose a conservative interpretation of results, using this as an exploratory method repeated over several brain regions to identify potentially important variables.

## Results

### Spatial Topography of TMS-evoked BOLD Signal

**Figure 2:**
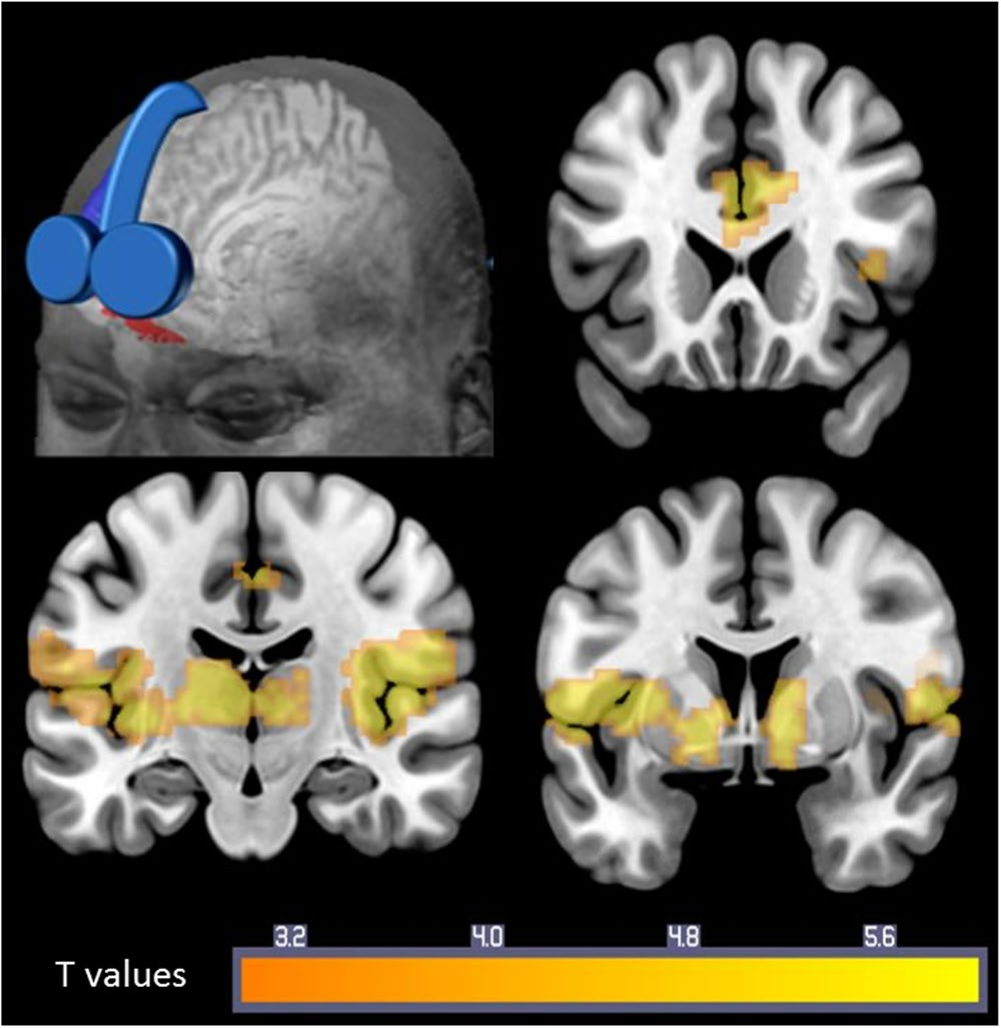
TMS-evoked BOLD response in cocaine users. Delivering TMS to the left frontal pole elicits robust responses in nucleus accumbens, caudate, putamen, anterior cingulate, insula, thalamus, superior temporal gyrus, and precuneus. All clusters Family Wise Error multiple comparison-corrected to p < 0.05.

Following VMPFC TMS, TMS-evoked BOLD signal increases were revealed in striatal regions, including the bilateral nucleus accumbens, bilateral caudate, and bilateral putamen, as well as in salience circuit regions, including bilateral anterior cingulate cortex (ACC) and anterior insula (cluster-level pFWE < 0.05, k ≥ 58; Fig. 2, Table 2). TMS-evoked BOLD signal increases were also revealed in the bilateral thalamus, bilateral superior temporal gyrus, bilateral lateral occipital, and precuneus. There were no regions with significant decreases following VMPFC stimulation. The average beta value for each of the ROIs is in Supplemental Figure S5.

**Table 2:** Brain regions significantly modulated by VMPFC TMS in cocaine users.

	L/R	Significant clusters	BA	MNI coordinates			Max t	Cluster-level p
				x	y	z		
*VMPFC stimulation: elevated BOLD signal*								
1 cluster, k = 7373	L	Anterior insula	38	−57	8	−1	10.04	<0.001
		Superior temporal gyrus						
	L	Middle temporal gyrus	21	−63	−52	11	10.02	
	L	Superior temporal gyrus	38	−54	5	−4	9.47	
	R	Posterior insula	48	36	−16	2	9.27	
		Anterior insula						
		Putamen						
	L	Middle temporal gyrus	37	−57	−58	2	9.15	
	L	Thalamus	—	−8	−16	2	6.15	
	R	Caudate	—	10	4	2	5.35	
		Nucleus accumbens						
	L	Caudate	—	−8	4	−2	4.55	
		Nucleus accumbens						
	R	Thalamus	—	11	−18	6	4.35	
	L	Putamen	—	−26	1	0	4.04	
1 cluster, k = 1727	R	Middle cingulate cortex	31	12	−31	50	6.34	<0.001
	L/R	Anterior cingulate cortex	24	8	5	38	6.08	
	L/R	Anterior cingulate cortex	24	−9	2	40	5.78	
	L/R	Anterior cingulate cortex	24	0	17	32	5.77	
	L	Middle cingulate cortex	31	−12	−34	44	5.25	
1 cluster, k = 58	L	Inferior occipital lobe	19	−42	−88	−1	4.67	0.039
	L	Inferior occipital lobe	18	−24	−97	−1	4.01	
	L	Middle occipital lobe	18	−33	−94	11	3.46	
	L	Middle occipital lobe	17	−24	−100	8	3.36	
	L	Middle occipital lobe	19	−48	−82	2	3.18	
		Middle temporal lobe						
*VMPFC stimulation: attenuated*								
*BOLD signal*		No significant clusters						

Abbreviations: VMPFC = ventromedial prefrontal cortex; TMS = transcranial magnetic stimulation; L = left; R = right; BA = Brodmann’s area; MNI = Montreal Neurological Institute; Max t = Maximum t-value; k = number of voxels; BOLD = blood oxygen-level dependent.

### Diffusion Tensor Imaging

The highest FA values were seen in white-matter tracts connecting the site of stimulation to the ACC, followed by the putamen and caudate (Supplemental Figure S2). Meanwhile, the lowest FA values were seen at the site of stimulation (FP1), a cortical region. Mean FA values (±SD) were as follows: FP1 = 0.360 (±0.030); FP1 to left putamen = 0.499 (±0.035); FP1 to left caudate = 0.425 (±0.033); FP1 to left ACC = 0.5256 (±0.044); FP1 to right ACC = 0.597 (±0.033). As expected, mean diffusivity (MD) in these tracts were higher in regions with low FA values (Supplemental Figure S3). Mean MD values (±SD) were as follows: FP1 = 0.670 × 10^−3^ mm^2^/s (±7.0 × 10^−5^); FP1 to left putamen = 0.662 × 10^−3^ mm^2^/s (±7.4 × 10^−5^); FP1 to left caudate = 0.669 × 10^−3^ mm^2^/s (±7.2 × 10^−5^); FP1 to left ACC = 0.649 × 10^−3^ mm^2^/s (±6.1 × 10^−5^); FP1 to right ACC = 0.647 × 10^−3^ mm^2^/s (±6.1 × 10^−5^).

### Voxel-Based Morphometry

Mean VBM GMV values were measured in units of voxel intensity (±SD) and were as follows: at the site of stimulation FP1 = 0.292 (±0.0301), in the left caudate = 0.327 (±0.0316) and right caudate = 0.324 (±0.0318), left putamen = 0.394 (±0.0405) and right putamen = 0.413 (±0.0372), left ACC = 0.384 (±0.0374) and right ACC = 0.335 (±0.0325), and left insula = 0.410 (±0.0314) and right insula = 0.419 (±0.0339) (results displayed in Supplemental Figure S4).

### Relationship between TMS-evoked BOLD signal and white matter integrity

**Figure 3:**
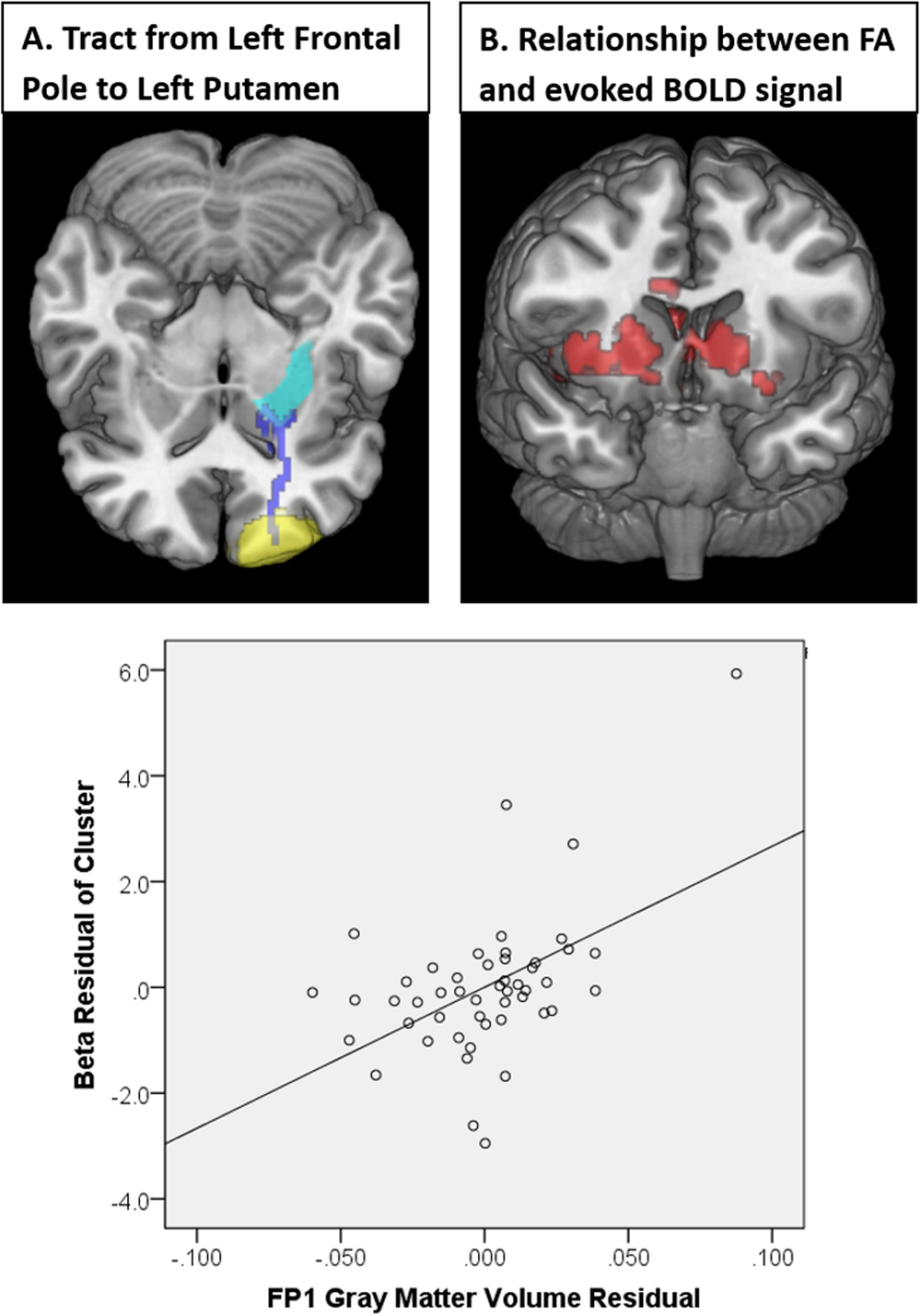
Relationship between white matter integrity and subcortical response to TMS. Using deterministic tractography, the tract between FP1 and the left putamen was isolated and the average FA values along the tract were compiled for each individual (**A**). As a group, there was a significant positive relationship between FA value along the tract and TMS-evoked BOLD signal in the striatum bilaterally (**B**). Individuals with higher tract integrity had a larger effect of TMS in these afferent targets of the frontal pole. A scatter plot shows the relationship between FP1 to putamen fractional anisotropy and cluster beta values after controlling for age, TMS pulses administered, diffusion protocol, and scalp to cortex distance (C).

The FA values along the FP1 to left putamen tract were positively associated with TMS-evoked BOLD signal in the bilateral putamen, and bilateral caudate (k = 682, cluster FWE corrected p < 0.005) (Fig. 3B). The partial correlation between FA in the FP1 to left putamen tract and TMS-evoked BOLD response in the striatal cluster (r = 0.441, p = 0.002) is shown in Fig. 3C. There was no significant relationship between FA values along the tract of FP1 to left caudate or ACC and TMS-evoked BOLD signal. MD measures in FP1 and along the tracts of interest were not significantly correlated with TMS-evoked BOLD response.

### Relationship between TMS-evoked BOLD signal and gray matter integrity

**Figure 4:**
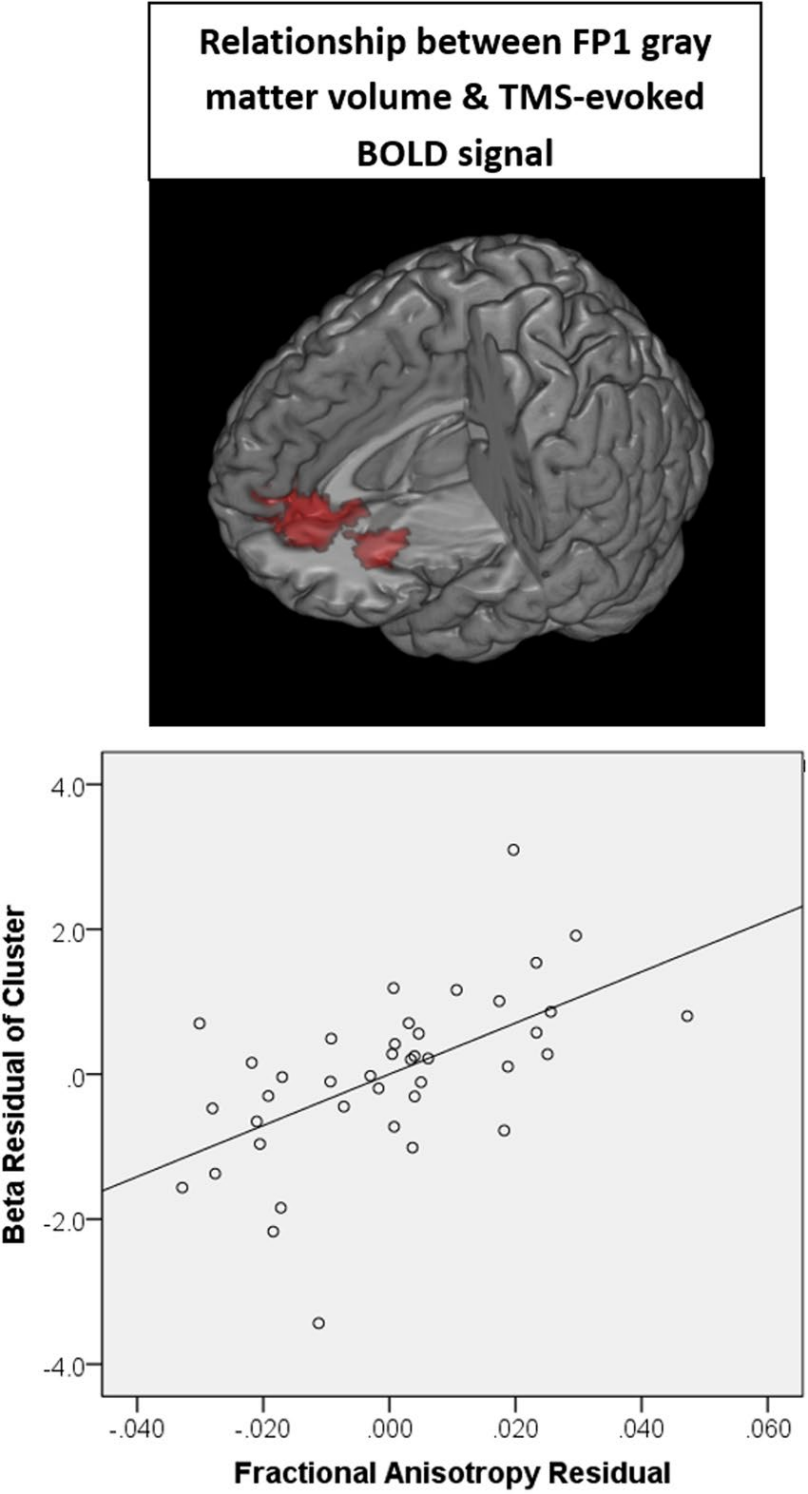
Relationship between gray matter integrity and subcortical response to TMS. Using voxel-based morphometry, the gray matter volume at the site of stimulation and afferent targets (see Supplemental Figure S1 for ROIs) was isolated. As a group, there was a significant positive relationship between the gray matter volume in the cortical site of stimulation (FP1) and TMS-evoked BOLD signal in the anterior-cingulate, as well as the orbitofrontal cortex. Individuals with higher gray matter volume had a larger effect of TMS in these cortical afferent targets. A scatter plot shows the relationship between FP1 gray matter volume and cluster beta values after controlling for participant age, TMS pulses administered and scalp to cortex distance (B).

The GMV at the site of stimulation (FP1) was positively associated with TMS-evoked BOLD signal in the bilateral ACC, medial orbitofrontal cortex, and superior frontal gyrus (k = 829, cluster FWE-corrected p < 0.005) (Fig. 4). The partial correlation between FP1 gray matter volume and TMS-evoked BOLD in this cluster (r = 0.575, p < 0.001) is shown in Fig. 4B. GMV in the left ACC was positively associated with TMS-evoked BOLD signal in a large cluster which included the left ACC (as well as the left middle cingulate, right anterior cingulate, and medial orbitofrontal cortex; k = 1309, cluster FWE corrected p < 0.001). GMV in the caudate, putamen, insula, and right ACC did not have a significant relationship to TMS-evoked BOLD signal.

### Relationship between TMS-evoked BOLD signal and clinical variables

Examining the variables that remained in each model following the stepwise linear regression process, certain variables showed up repeatedly as significant predictors (Supplemental Table S1). BDI remained in the models as an important predictor for each of the brain regions, with the exception of FP1. BDI had a positive linear relationship with all regions, indicating that individuals with a higher BDI score had greater striatal and limbic TMS-evoked BOLD response. Scalp-to-cortex distance and TMS threshold also appeared to be important predictors of TMS-evoked BOLD response, as they remained in the models for seven of the nine brain regions. Scalp-to-cortex distance had a negative linear relationship with each region, except for FP1 and the left putamen. TMS threshold had a positive linear relationship with all regions, except the left and right putamen. The remaining variables appeared more sparingly in this analysis. This does not necessarily indicate that the other variables were not good predictors, but that they may have a more isolated relationship with TMS-evoked BOLD response that would warrant further investigation.

## Discussion

Transcranial magnetic stimulation (TMS) is now a widely-used neuromodulation approach for multiple clinical populations with psychiatric or neurologic illness. One primary feature of TMS that distinguishes it from other neuromodulation techniques is that through direct modulation of the cortex, it is possible to indirectly modulate subcortical regions monosynaptically connected to the target^23–25^. This general principle of TMS assumes that the structural architecture of the brain is intact. In this study we present, for the first time, a large set of data which demonstrates that the effects of TMS on cortical and subcortical activity are dependent upon the structural architecture of white and gray matter in the brain. Specifically, single pulses of TMS applied to the left VMPFC of cocaine users are significantly more likely to evoke changes in BOLD signal in the striatum in individuals that have higher values of FA along the tract that connects the VMPFC to the striatum. Additionally, higher levels of gray matter volume in the vicinity of the coil are associated with a higher BOLD signal in the ACC and OFC. ***These data represent the first set of empirical evidence that the efficacy of TMS as a tool to modulate subcortical regions is dependent upon the structural integrity of the pathway from the cortical target****.* In addition, these findings suggest that future studies should use structural architecture as a covariate in their analyses, as the future clinical utility of TMS is dependent on considering structural architecture as a tool for patient-specific dosing.

Given that traditional figure-of-eight TMS coils only have stimulation penetration depths of approximately 20 mm^26^, subcortical modulation by TMS depends on functional connectivity between cortical targets and their subcortical afferents. This is likely dependent on an intact structural pathway between the  regions. Both clinical and preclinical studies have shown structural connectivity measures, such as white matter integrity, to be highly related to functional connectivity^27,28^. There is also considerable evidence that cocaine users have significantly lower white matter integrity within and between frontal-striatal-thalamic regions^6,7,29,30^. This loss of white matter is non-trivial to TMS treatment development in this population, as the brain response to TMS is likely dependent on the integrity of these cortical-subcortical functional and structural connections. Yet, despite the importance of white matter integrity, its role in TMS efficacy has largely been overlooked.

To our knowledge, this is the first study to investigate brain structure as it relates to TMS-evoked BOLD response in substance-using individuals. Consistent with previous studies, we found a range of white matter integrity and gray matter volumes across frontal-striatal regions in cocaine users^31,32^. We hypothesized that lower white matter integrity would impede responses to VMPFC TMS in the frontal-striatal-thalamic circuit. We found that white matter integrity between the site of TMS stimulation and the subcortical target is  critical to generating subcortical activity. This suggests that in many patient populations with white matter disease (e.g. alcoholism, drug abuse, multiple sclerosis, dementia) the TMS-induced engagement of the striatum will be greatest in individuals with higher levels of white matter integrity.

However, there were several white matter tracts (e.g. frontal pole-caudate) for which there was no significant relationship between FA value and TMS-evoked BOLD signal, despite the range of FA values identified. It is possible that for some individuals this phenomenon may reflect what is known as cognitive or brain reserve. Brain reserve can be defined as the brain’s resilience, or its ability to cope with increasing damage while still functioning adequately^33–35^. For instance, in some patients with Alzheimer’s disease, there is a discrepancy between the degree of Alzheimer’s disease neuropathology and the clinical manifestations of the disease^36^. Some patients whose brains have extensive Alzheimer’s disease pathology, clinically show little to no manifestations of the disease. These patients with a higher brain reserve show greater thresholds before clinical deficits appear^37^. Likewise, our data may suggest that some cocaine users may need to exhibit substantial structural white matter loss before showing functional implications (i.e. impairments in processing between cortical and subcortical regions) that would affect TMS-evoked responses.

Interestingly, we also found that gray matter volume at the site of stimulation is critical for activating regions within the salience network. VMPFC gray matter volume reductions have been shown among cocaine users^38–40^. Therefore, it is important for investigators to consider cortical volume measurements in addition to scalp-to-cortex distance. However, previous studies also indicate that gray matter volumes can recover following sustained abstinence^41^. This suggests that efficacy of TMS may be greater after a period of abstinence or prior to chronic cocaine administration.

Another interesting observation from these data is that while the white matter integrity from the site of stimulation to subcortical afferents accounts for a significant percent of the observed variance, there is still variance in the regional BOLD signal that is not accounted for by structure alone. Specifically, the logic of the present study would suggest that the area whose white matter tract has the greatest FA would also have the greatest BOLD response. This is, in fact, verified with the ACC. The local maxima of BOLD signal in the left ACC, caudate, and putamen peak at 6.08, 4.55, and 4.04 respectively (Table 2). The FA value along the tract for the left ACC, caudate, and putamen are 0.52, 0.50, and 0.43 respectively (Supplemental Figure S2). So, while the ACC has the highest BOLD signal and the highest FA (consistent with the logic of the present study) the caudate and putamen appear in a different order. Interestingly, if we extract beta values from ROIs for the BOLD data, the putamen has the highest average BOLD beta value (0.79), the ACC is very close (0.67), yet the caudate is, again, near the bottom (0.18) (Supplemental Figure S5). Taken together these data indicate that while FA has a significant contribution to the direct effects of TMS on evoked BOLD signal, there are likely other variables that contribute to BOLD signal elevations in an interleaved TMS/MRI paradigm. This is well demonstrated in Supplemental Figure S5 wherein the BOLD signal in left auditory cortex (positive control ROI) is greater than any other ROIs (likely due to the sound of TMS). Future studies using sham-TMS paradigms are necessary to examine the direct and indirect effects of TMS on evoked BOLD signal.

Furthermore, we also investigated other common sources of variance that we hypothesized might influence TMS-evoked BOLD activity. To name a few, sex^42^, duration of use^43^, and use severity^44^ have been associated with differing patterns of drug cue-related activation in substance users. Taking advantage of our large heterogeneous sample, the present study sought to identify clinical and demographic factors that might influence the brain’s response to VMPFC TMS. Examining the variables that remained in each model following the stepwise linear regression process, certain variables showed up repeatedly as significant predictors. These included BDI scores, scalp-to-cortex distance, and TMS threshold. One potential explanation for the relationship with BDI scores is that depressive symptoms have been associated with attentional bias for negative stimuli^45^. While interleaved TMS/fMRI may only be experienced as a mildly unpleasant stimulus among healthy subjects, depressed subjects tend to shift their attention toward negative stimuli. Furthermore, attentional bias to negative stimuli among depressed subjects has previously been associated with an increased activation of striatal and salience network regions^46^.

Factors including sex, days of cocaine use in the last month, marijuana use, nicotine use, AUDIT scores, and State-Trait Anxiety scores were not significantly related to TMS-evoked BOLD response in the frontal-striatal-thalamic circuitry. This suggests that, other than the structural architecture of the brain as described above, TMS to the VMPFC is likely to induce reliable and consistent neural responses despite inherent individual variability in these factors. This is encouraging given the highly-diverse patient populations expected in future clinical trials.

There are a few limitations of the present work that deserve consideration. As with most studies of cocaine-dependent individuals, 43 of the 49 participants were nicotine users, and 35 were marijuana users. This diverse but representative sample precludes our ability to state that this is a property specific to cocaine, but increases the likelihood that these findings are a more general principle of the neurobiology of TMS rather than something specific to a given category of drug use. Further studies in populations with known demyelinating diseases or focal neurodegenerative disease are needed to fully explore the transdiagnostic relevance of this study. Additionally, this manuscript represents the amalgamation of three different data sets which had different numbers of TMS pulses. Although this was accounted for in the analysis, it is important to note that there may have been other systematic aspects of these three studies which may influence the repeatability of the results. Although this is a large data set it is not large enough to be sufficiently powered to evaluate and compare each group independently.

Moving forward, these results suggest that white matter integrity and gray matter volume should be taken into account as sources of variance when developing TMS as a treatment tool for populations with known changes in neural structure. Although the results of this study are limited to cocaine users, the consistency of these empirical data with current theories regarding the mechanism of action through which TMS modulates the striatum, suggest that this may be a transdiagnostic feature that should be taken into account when delivering TMS to other clinical populations.

### Data Availability Statement

Any supporting data needed or requested by Editorial Board Members and/or referees are or will be made available.

### ClinicalTrials.gov Identifier

NCT02939352; Registration date: September 28, 2015.

## Electronic supplementary material

Supplementary Tables and Figures
