# Author Correction: Gray and white matter integrity influence TMS signal propagation: a multimodal evaluation in cocaine-dependent individuals

**DOI:** 10.1038/s41598-018-24113-8

**Published:** 2018-04-20

**Authors:** Tonisha E. Kearney-Ramos, Daniel H. Lench, Michaela Hoffman, Brittany Correia, Logan T. Dowdle, Colleen A. Hanlon

**Affiliations:** 1 Department of Psychiatry, Medical University of South Carolina, Charleston, South Carolina USA; 2 Department of Neurosciences, Medical University of South Carolina, Charleston, South Carolina USA; 3 Center for Biomedical Imaging, Medical University of South Carolina, Charleston, South Carolina USA; 4 Ralph S. Johnson VA Medical Center, Charleston, South Carolina USA

Correction to: *Scientific Reports* 10.1038/s41598-018-21634-0, published online 19 February 2018

In Figures 3 and 4, the scatter plots are incorrectly shown the wrong images. The correct Figures 3 and 4 appear below as Figures 1 and 2.

**Figure 1:**
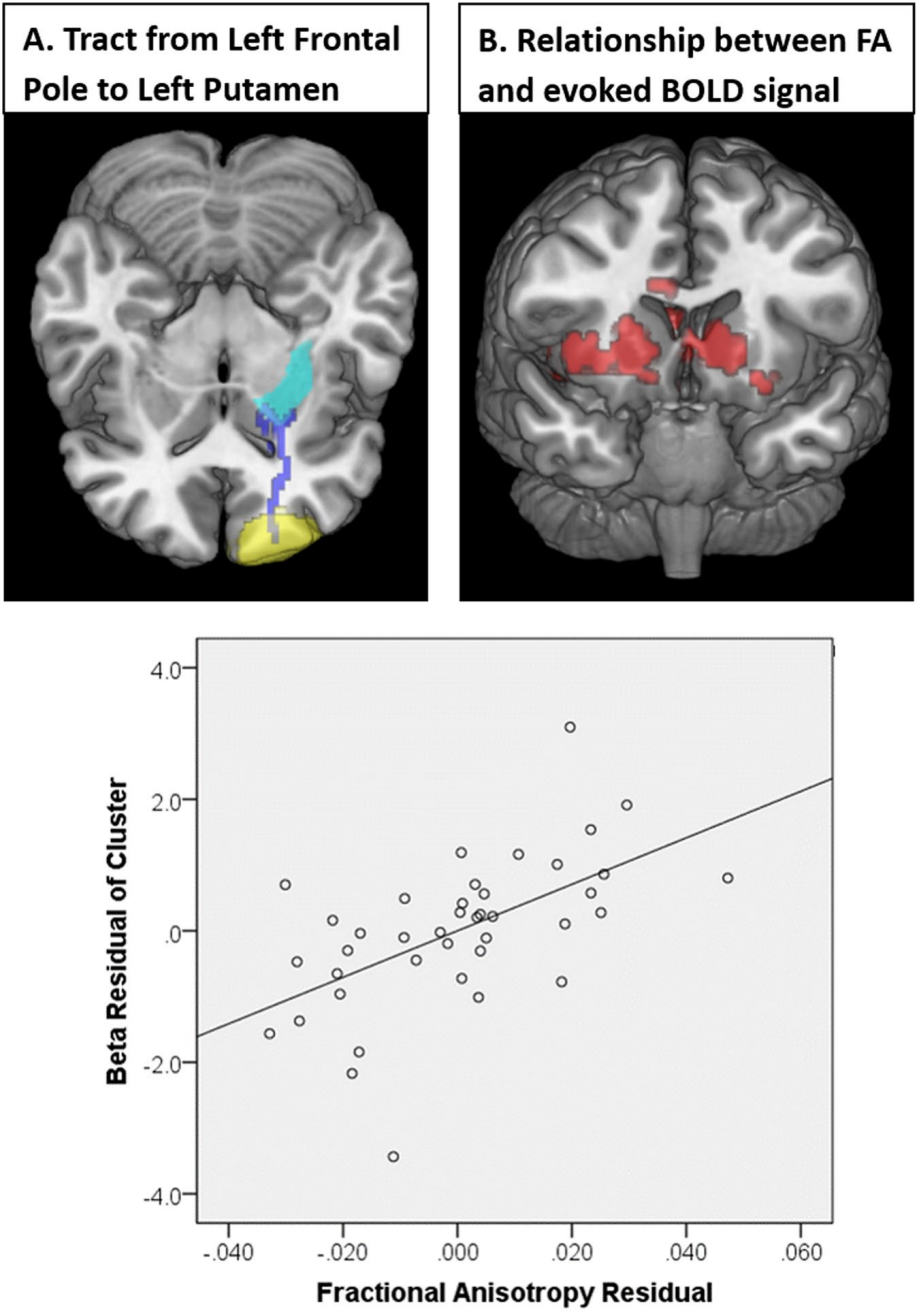
Relationship between white matter integrity and subcortical response to TMS. Using deterministic tractography, the tract between FP1 and the left putamen was isolated and the average FA values along the tract were compiled for each individual (**A**). As a group, there was a significant positive relationship between FA value along the tract and TMS-evoked BOLD signal in the striatum bilaterally (**B**). Individuals with higher tract integrity had a larger effect of TMS in these afferent targets of the frontal pole. A scatter plot shows the relationship between FP1 to putamen fractional anisotropy and cluster beta values after controlling for age, TMS pulses administered, diffusion protocol, and scalp to cortex distance (C).

**Figure 2:**
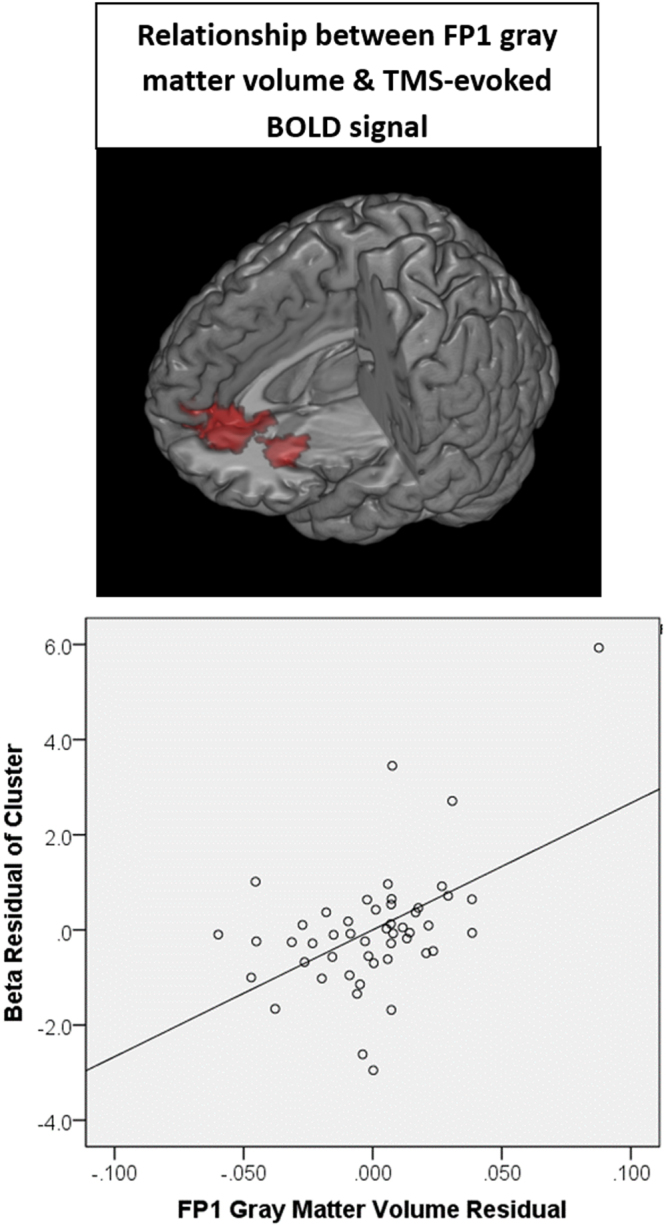
Relationship between gray matter integrity and subcortical response to TMS. Using voxel-based morphometry, the gray matter volume at the site of stimulation and afferent targets (see Supplemental Figure S1 for ROIs) was isolated. As a group, there was a significant positive relationship between the gray matter volume in the cortical site of stimulation (FP1) and TMS-evoked BOLD signal in the anterior-cingulate, as well as the orbitofrontal cortex. Individuals with higher gray matter volume had a larger effect of TMS in these cortical afferent targets. A scatter plot shows the relationship between FP1 gray matter volume and cluster beta values after controlling for participant age, TMS pulses administered and scalp to cortex distance (B).

