# Supplementary material for: Gray and white matter integrity influence TMS signal propagation: a multimodal evaluation in cocaine-dependent individuals
Source: Sci Rep. 2018 Feb 19;8:3253. doi: 10.1038/s41598-018-21634-0 (PMC5818658; doi:10.1038/s41598-018-21634-0)
Supplement: Supplementary file 1 — Supplementary Tables and Figures [file 41598_2018_21634_MOESM1_ESM.pdf]

Title: Gray and white matter integrity influence TMS signal propagation: a multimodal evaluation in cocaine-dependent individuals

Tonisha E. Kearney-Ramos, Daniel H. Lench, Michaela Hoffman, Brittany Correia, Logan T. Dowdle, and Colleen A. Hanlon

Supplemental Materials

Table S1. Stepwise Multiple Linear Regression Results

			FP1		L caudate		R caudate		L putamen		R putamen	
			β	p	β	p	β	p	β	p	β	p
Intercept	#	%	0.55	0.05	0.35	0.04	0.24	0.37	0.94	0.00	0.88	0.00
BDI	5	56%			0.52	0.00	0.60	0.00	0.54	0.02	0.88	0.00
Scalp Distance	4	44%			-0.29	0.15	-0.32	0.13			-0.42	0.04
TMS Threshold	4	44%	0.41	0.15	0.50	0.01	0.55	0.01				
Age First Use	3	33%	0.41	0.15					0.26	0.17	0.46	0.02
State Anxiety	2	22%							0.39	0.09		
Years of Use	0	0%										
AUDIT	1	11%					-0.24	0.18				
Fagerstrom	1	11%					-0.28	0.14				
Gender (Male)	1	11%					0.51	0.18				
R Squared			0.12		0.33		0.43		0.42		0.43	
Adjusted R2			0.07		0.27		0.32		0.38		0.38	
N			39		39		39		39		39	

The betas (β) and p-values (p) reflect the contribution of each variable to the regression model for that brain region. L occipital and L auditory were included as negative controls. Abbreviations and notations: L = left; R = right; # = number of models in which that variable significantly contributed; % = percent of all models in which that variable significantly contributed; BDI = Beck's Depression Inventory; Scalp Distance = scalp-to-cortex distance (mm); State Anxiety = State-Trait Anxiety Inventory; Years of Use = years of cocaine use; AUDIT = Alcohol Use Disorders Identification Test; Fagerstrom = Fagerstrom Test for Nicotine Dependence

Table S1. Stepwise Multiple Linear Regression Results…continued

	L ACC		R ACC		L insula		R insula		L occipital		L auditory	
	β	p	β	p	β	p	β	p	β	p	β	p
Intercept	0.91	0.00	0.62	0.00	1.30	0.00	1.71	0.00	-0.46	0.31	1.22	0.01
BDI	0.51	0.02	0.60	0.00	0.56	0.02	0.51	0.10	0.77	0.02		
Scalp Distance	-0.42	0.05	-0.37	0.08	-0.56	0.03	-0.70	0.04				
TMS Threshold	0.56	0.01	0.35	0.10	0.47	0.04	0.49	0.09			0.42	0.15
Age First Use			0.30	0.09								
State Anxiety	0.30	0.16			0.49	0.04	0.74	0.01			0.94	0.00
Years of Use					0.39	0.10	0.49	0.11				
AUDIT												
Fagerstrom									-0.59	0.07	-0.63	0.03
Gender (Male)									1.86	0.00	1.22	0.05
R Squared	0.42		0.35		0.51		0.47		0.32		0.39	
Adjusted R2	0.35		0.28		0.44		0.39		0.26		0.32	
N	39		39		39		39		39		39	

The betas (β) and p-values (p) reflect the contribution of each variable to the regression model for that brain region. L occipital and L auditory were included as negative controls. Abbreviations and notations: L = left; R = right; # = number of models in which that variable significantly contributed; % = percent of all models in which that variable significantly contributed; BDI = Beck's Depression Inventory; Scalp Distance = scalp-to-cortex distance (mm); State Anxiety = State-Trait Anxiety Inventory; Years of Use = years of cocaine use; AUDIT = Alcohol Use Disorders Identification Test; Fagerstrom = Fagerstrom Test for Nicotine Dependence

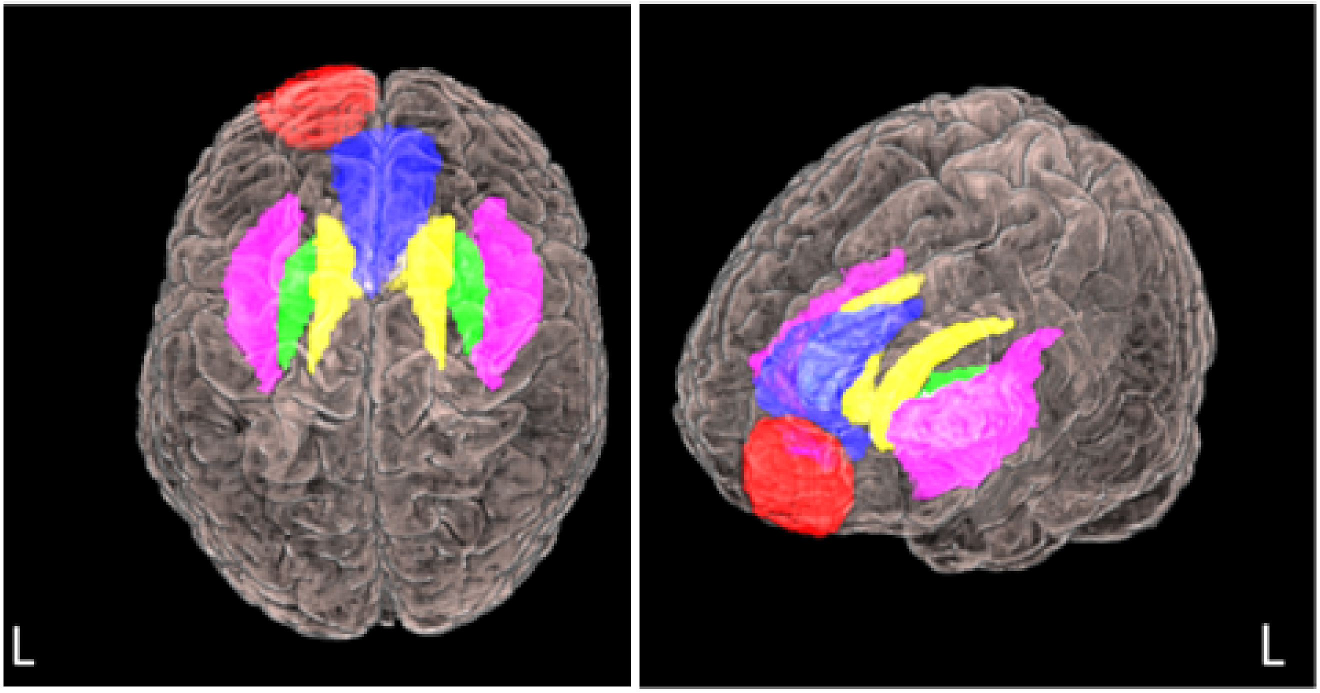

Supplemental Figure S1. Regions-of-interest (ROIs) used for the VBM analysis and seeds for the DTI analysis. ROIs were selected from the standard Automated Anatomical Labeling (AAL) Atlas. (AAL: caudate (Caudate_L, Caudate_R; yellow), putamen (Putamen_L, Putamen_R; green) insula (Insula_L, Insula_R; pink), and anterior cingulate cortex (left and right Cingulum_Ant; blue). Additionally a custom ROI was created for FP1 using a cortical location previously described [47], wherein a spherical ROI was created with a radius of 20mm and masked with an MNI brain mask (FP1; red). FP1 (red) was used as the starting seed, while the other ROIS were end seeds in the DTI analysis.

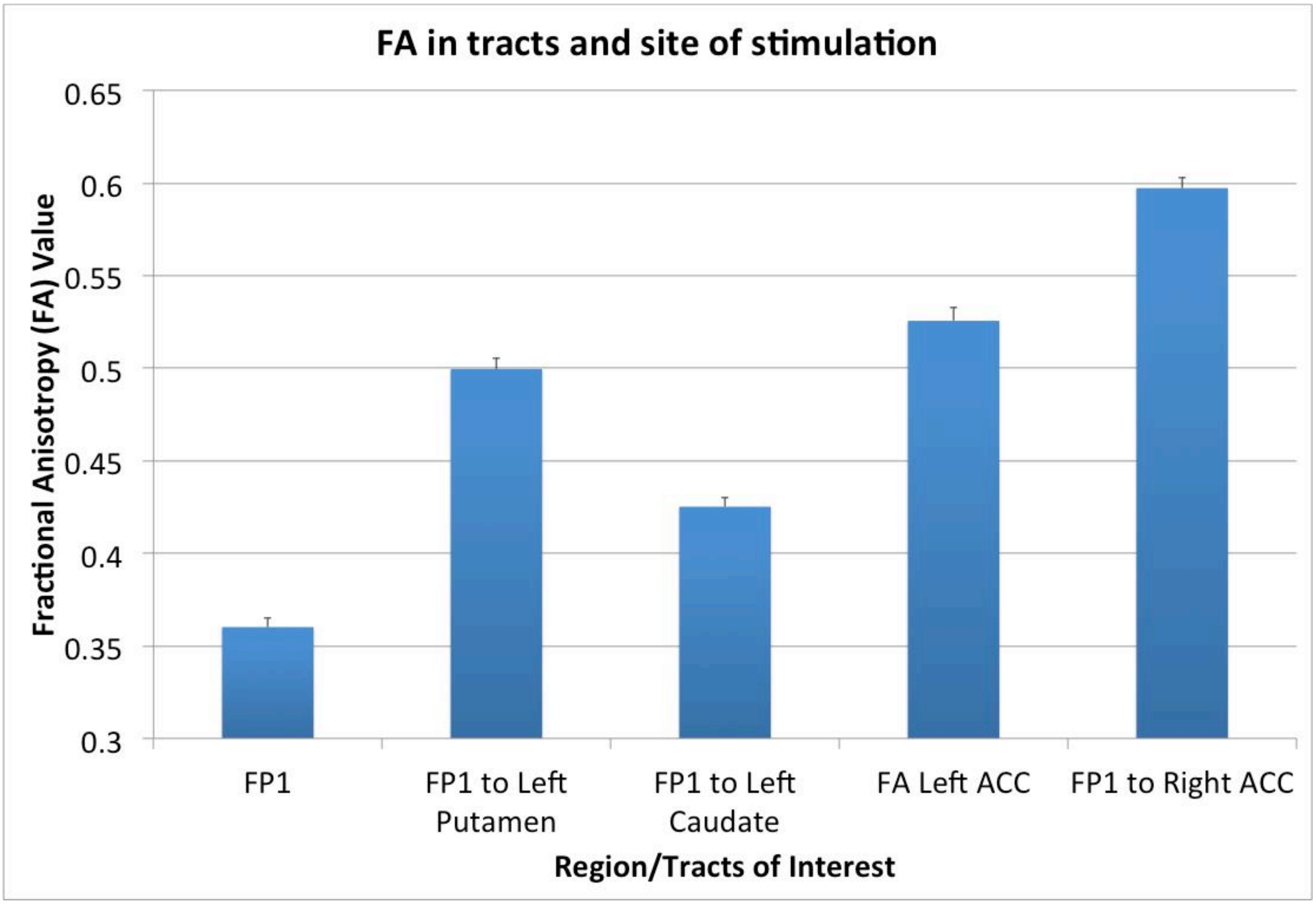

Supplemental Figure S2. Mean fractional anisotropy (FA) measures (± standard error of mean) extracted from each cocaine subject's skeletonized FA map. Regions of interest for extraction included the site of stimulation (FP1), and tracts from the site of stimulation to left putamen, left caudate, left anterior cingulate cortex (ACC) and right ACC.

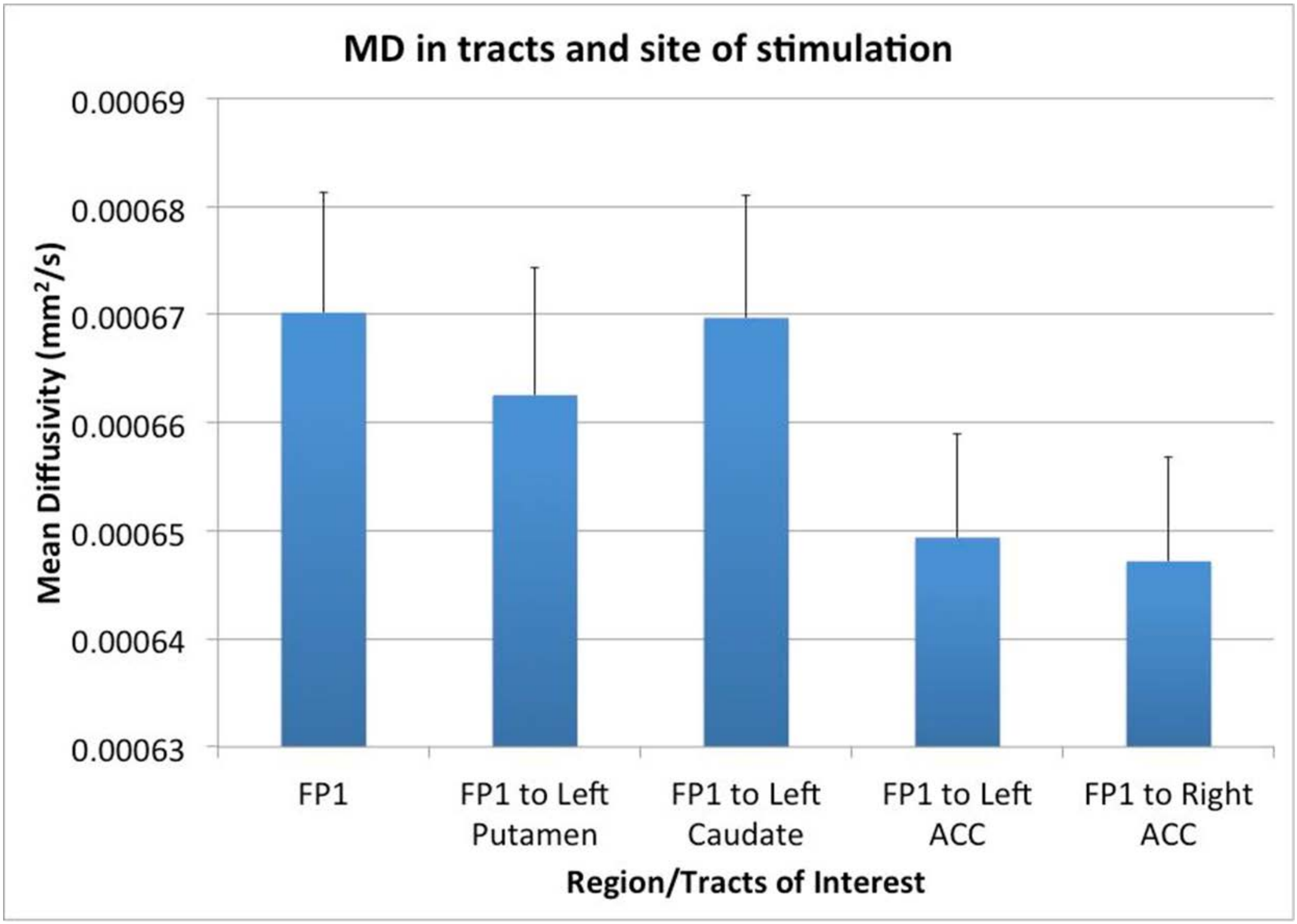

Supplemental Figure S3. Mean mean diffusivity (MD) measures (± standard error of mean) extracted from each cocaine subject's skeletonized MD map. Regions of interest for extraction included the site of stimulation (FP1), and tracts from the site of stimulation to left putamen, left caudate, left anterior cingulate cortex (ACC) and right ACC.

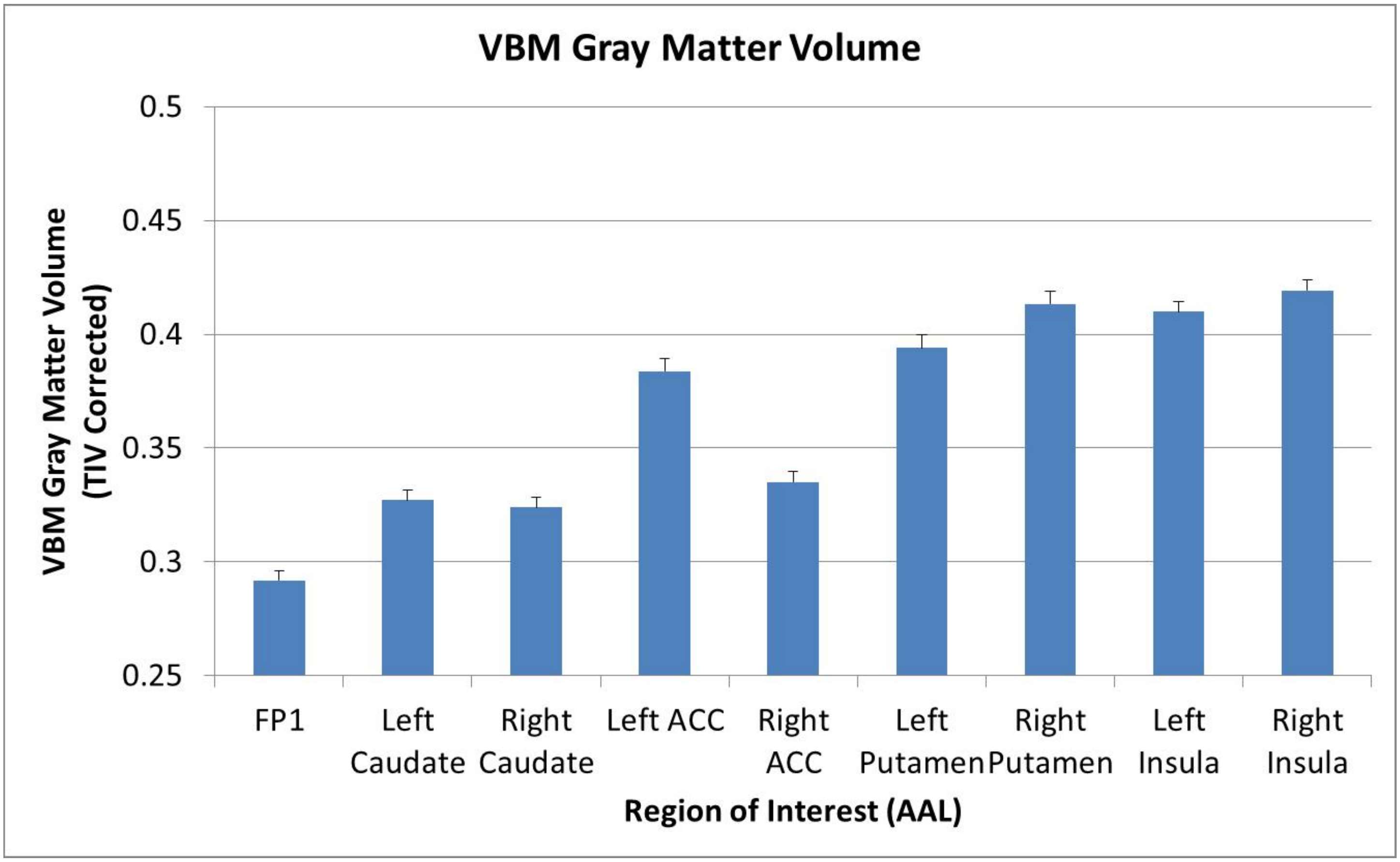

Supplemental Figure S4. Mean voxel-based morphometry (VBM) gray matter volume values (± standard error of mean) extracted from the site of stimulation (FP1) and various salience and striatal regions. To correct for global effects of head size, VBM gray matter volume was divided by each subject's total intracranial volume (TIV: CSF, WM, GM). ACC = anterior cingulate cortex; AAL = Automated Anatomical Labeling.

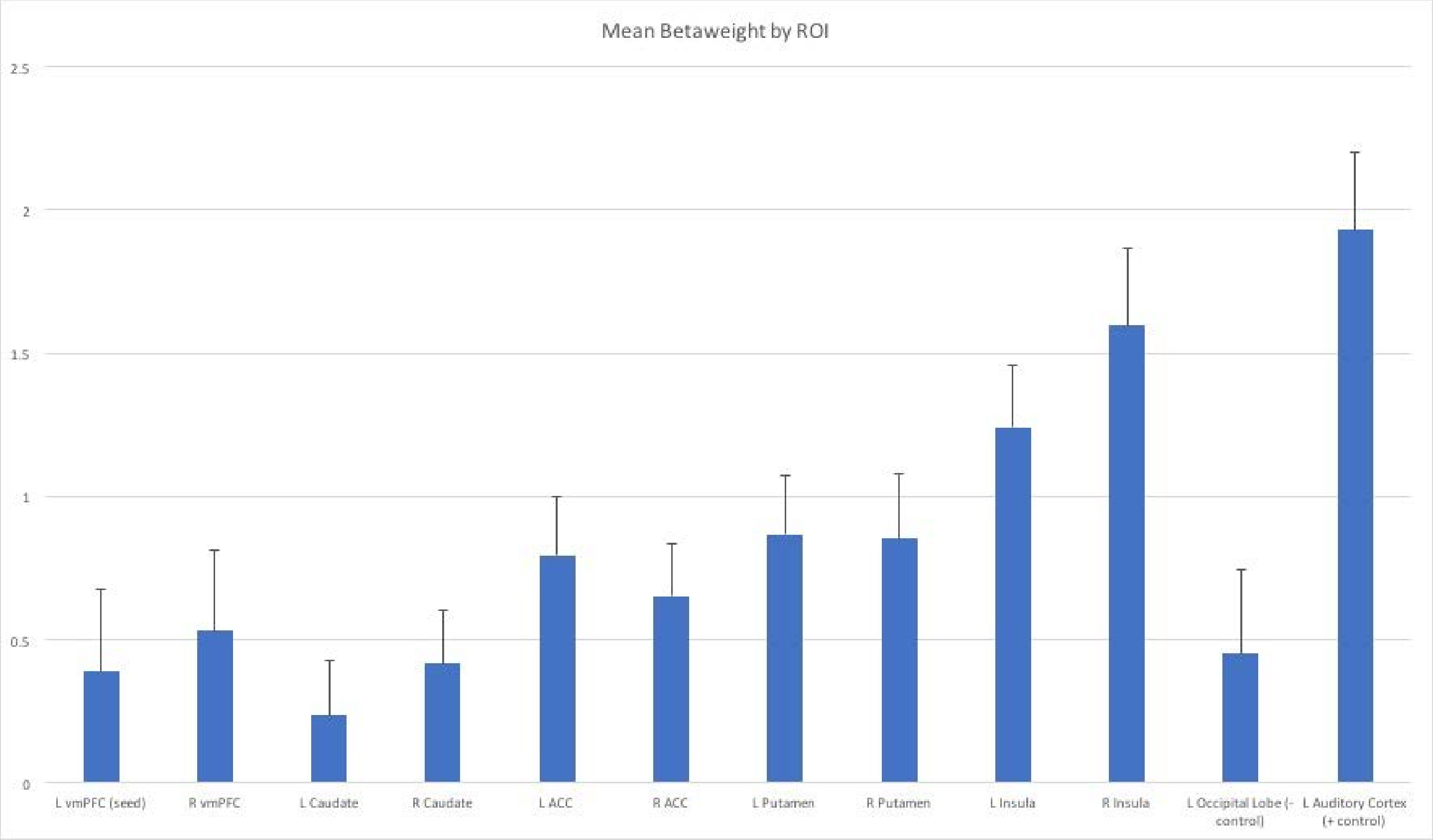

Supplemental Figure S5. Mean BOLD signal beta values (± standard deviation) extracted from bilateral ventromedial prefrontal cortex (VMPFC), and various salience and striatal regions. L = left; R = right; ACC = anterior cingulate cortex.
